# Impacts of Social Environments on Neighborhood Depression Incidence: Fully Accounting for Spatial Effects

**DOI:** 10.3390/ijerph23020247

**Published:** 2026-02-16

**Authors:** Peter Congdon, Esmail Abdul-Fattah

**Affiliations:** 1 Department of Geography and Environmental Science, Queen Mary University of Londo, London E1 4NS, UK; 2 Extreme Computing Research Center, King Abdullah University of Science and Technology, Thuwal 23955-6900, Saudi Arabia

**Keywords:** depression, neighborhood, crime, collinearity, cohesion, urbanicity, heterogeneity, spillover, non-stationarity

## Abstract

Neighborhood variations in depression, an important aspect of the overall mental health burden, have been linked both to environmental context (e.g., area crime, neighborhood cohesion), and to area socio-demographic composition. Previous models seeking to explain such spatial variations in mental health, such as those based on Bayesian disease mapping, follow a standard approach defined by: spatially stationary effects of area predictors; predictor effects neglecting potential spatial spillover; and a spatially structured residual to account for unmodelled spatial dependencies. In a study of depression incidence in England neighborhoods, we consider the gains from an alternative strategy, allowing nonstationary environmental impacts; spillover effects of environmental factors, and a non-stationary spatial intensity. We focus particularly on impacts of socio-behavioral environments, namely neighborhood cohesion and crime. We find these to be major influences on neighborhood depression incidence, and also find major gains in model performance by explicitly considering non-stationarity and spillovers. Allowing context heterogeneity, varying spatial intensity and spillover are shown to enhance the impacts of socio-behavioral environments on depression incidence, and such findings have broader relevance to disease mapping regression. Public health policy framing may therefore need to be tailored to locally specific environmental impacts, and to inter-agency collaboration across arbitrary boundaries.

## 1. Introduction

Neighborhood variations in depression are an important aspect of the overall mental health burden, and typically co-morbid with other mental illnesses such as psychosis. Such variations, or those in common mental disorders more generally, have been less systematically explored using advanced spatial modelling approaches, though available studies [1,2] show major influences of area socio-demographic composition, ethnic mix, and contextual environmental factors, such as area crime, urbanicity, and neighborhood social cohesion. For guiding public health intervention and assessment for resourcing, a small area neighborhood perspective is increasingly recognized as central [3], as place effects are acknowledged to play a role in disease variations as well as individual risk factors. Thus, meta-analysis [4] shows significant impacts of neighborhoods on depressive symptoms, controlling for individual attributes (such as age, income, and ethnicity). The present study adopts this small-area perspective to examine how socio-behavioral environments, particularly cohesion and crime, shape neighborhood-level depression incidence in England.

Preventive interventions for tackling depression are important as it is not only a clinical/individual issue, but a major contributor to population-level disease burdens [5]. More severe and recurrent subtypes drive demand for mental healthcare, especially at primary care level [6], making neighborhood-level variation in incidence directly relevant to local service planning.

We focus in the present study on place effects in neighborhood depression, namely impacts of socio-behavioral environments. Such neighborhood factors are potentially modifiable and therefore relevant for community- and place-based public health strategies [7]. The study [7], p. 56 highlights the relevance of modifiable social and environmental determinants to the onset (or exacerbation) of mental health problems, while noting the relatively late attention (compared to biomedical influences) paid to them.

Hence establishing robustly the impacts of environmental determinants on mental health outcomes is important. In particular, we consider the role in explaining neighborhood depression of cohesion and crime [8], how these impacts operate spatially, and so determine which areas are at highest risk.

It has been argued [9] that existing studies of depression over-emphasize the relevance of socio-economic composition and neglect the role of contextual neighborhood characteristics, both objective and subjective (e.g., actual and perceived crime levels). Thus [9], p. 41 states “failure to control for nesting of people within geographic space […], and an almost exclusive focus on neighborhood socioeconomic status […] limit understanding of the ways in which neighborhood characteristics influence depressive symptoms”.

### 1.1. Full Accounting for Spatial Effects

As well as taking fuller account of health impacts linked to socio-environmental contexts, we also consider associated issues in appropriate modelling of spatial impacts, and in developing suitably disaggregated indicators. Models for spatial variations in mental health, such as those based on Bayesian disease mapping [10,11], follow the standard disease mapping approach [12,13], namely: spatially homogenous effects of area predictors; predictors defined without regard to possible spillover effects; and a spatially structured residual random effect to account for all unmodelled spatial dependencies.

We instead here assess gains from considering impacts of spatial structure and spatial dependence more comprehensively, specifically, improved model fit, more accurate estimation of predictor effects, and spatially differentiated findings that may inform locally tailored policy responses. A preliminary step is the development of a measure of neighborhood cohesion, as this has been shown as a significant environmental influence on neighborhood mental health, and relevant signifiers (e.g., community belongingness) are not measured for neighborhoods in Censuses [8].

Assessing the role of spatial structure in establishing environmental impacts is then done in three major ways.

First, we consider spatial spillovers in environmental influences. The importance of spillover is acknowledged in causal approaches to assessing impacts of neighborhood exposures [14]. It is also recognized in spatial linear regression methodologies, including regression on spatially lagged predictors, and known as spatial Durbin regression [15]. An example of these two approaches to overlap is provided in studies of pollution impacts by Giffin et al. [16] using a causal method, and by Sarrias and Molina-Varas [17], using spatial Durbin regression.

Spillover may also characterize impacts of social environments (e.g., cohesion, crime), access environments (e.g., exercise access) and so on, as these are not defined spatially by the arbitrary administrative boundaries of the neighborhoods typically used in ecological disease studies. For example, residents living nearer neighborhood boundaries are likely to experience spillover effects of environments in adjacent areas. Regarding neighborhood crime rates, Graif [18] mention that “despite the rapidly growing ecological-level evidence indicating geographic spillover effects on crime, research on neighborhood effects […] has treated neighborhoods as if they were isolated islands, independent of their surroundings”.

On conceptual grounds, spatial spillover would be anticipated as most relevant for contextual environmental influences. A compositional effect is inherently an effect of the socio-demography of the local population, whereas a contextual effect from environmental influences is spatially unbounded in impact, spilling over administrative boundaries.

Second, we explicitly consider context heterogeneity (spatial non-stationarity), particularly regarding environmental influences. As discussed in [19], p.112 “much of the observed spatial correlation in residuals that is frequently observed […] applied to spatial data results from applying a global model to a non-stationary process”. Non-stationarity can often be summarized in terms of spatial regimes, defined by Vidoli and Benedetti [20] as “an aggregation of neighboring units that are homogeneous in functional terms or that share the same relationship between a dependent variable and some covariates”. These have been infrequently considered in spatial epidemiology, though see Sridharan et al. [21] for an exception.

Non-stationarity in neighborhood depression has been analyzed using frequentist geographically weighted (GWR) regression [22,23]. Drawbacks with the usual application of GWR are mentioned by [24]. Here we use Bayesian approaches [25] to spatially varying coefficients (SVC) that have been shown to have advantages in analyzing spatial data. For example, these include full posterior summaries regarding spatial random effects and can also be applied with variances themselves treated as non-stationary.

Thus, we allow for variations in the precision of spatial random effects, both for spatially varying coefficients and for the spatial residual. The study [26] considered such heteroscedasticity just for spatial residuals, because spatial effects may be inherently less variable in some parts of the overall region (domain) being considered. We extend this approach here to heteroscedasticity in SVC effects.

Under the conditional autoregressive (CAR) spatial random effects approach of Besag et al. [27] commonly used in the standard Bayesian disease mapping model, spatial variability, as measured by a precision parameter, is assumed constant over the domain. This assumption may be restrictive for a large domain with many small areas. We accordingly use multiple precision parameters, expressing different intensities of spatial dependence in different sub-regions. We apply a penalized complexity prior [28] to varying precision parameters to prevent overfitting.

Heteroscedasticity in spatial effects has been considered before in Bayesian applications e.g., [29] but existing approaches may involve many weakly identified parameters. Spatial volatility models are quite widely applied in spatial econometric applications using simultaneous autoregressive (SAR) models applied to continuous responses e.g., [30] under a linear normal assumption. However, these methods do not transfer straightforwardly to geographic disease applications, typically with count responses.

A further consideration is that environmental impacts on depression may not be uniform across space. Urban and rural areas differ in social connectivity, crime exposure, and access to services, while English regions show distinct socio-economic profiles. We therefore also examine whether the effects of cohesion and crime vary by region and urbanicity, with implications for regionally tailored public health responses.

### 1.2. Study Aims and Objectives

This cross-sectional ecological study examines depression incidence across 6856 England neighborhoods, with both methodological and substantive public health objectives.

Methodologically, since observed cohesion indicators are unavailable at neighborhood level, we seek to derive small-area cohesion estimates using cross-scale modelling methods [31,32]. This involves disaggregating data to the 6856 neighborhoods from 296 larger areas (English local authorities). We further aim to evaluate whether allowing for spatial non-stationarity (context heterogeneity and heteroscedasticity) and spillover effects improves model fit compared to standard disease mapping approaches, and whether these extensions alter estimated effect sizes for environmental predictors.

Substantively, we aim to quantify the relative contributions of socio-behavioral environments (cohesion, crime) and socio-demographic composition in explaining neighborhood variation in depression incidence, and to identify which areas are at highest risk. We hypothesize that crime and low cohesion are associated with higher depression incidence, and that models accounting for non-stationarity and spillover will reveal stronger total environmental effects than conventional approaches. Such spatially differentiated findings may inform locally tailored public health planning and resource allocation.

## 2. Data and Methods

### 2.1. Outcome Data

We seek to explain neighborhood variations in the incidence of clinically diagnosed depression (among people over 18) for N = 6856 Census defined neighborhoods, known as Middle Level Super Output Areas (MSOAs). These provide entire coverage of England, one of the UK nations. These data are obtained from the Quality Outcomes Framework (QOF), which records chronic disease prevalence in UK primary care [33,34]. There were 714,592 new depression cases across England in the year considered (2023/24), with an incidence rate (among adults) of 16.3 per 1000. Expected MSOA cases are based on age rates for depression from the Global Burden of Disease site (https://www.healthdata.org/research-analysis/gbd; accessed on 3 February 2025) applied to MSOA populations.

There are considerable spatial inequalities in incidence, going beyond what variations in socio-demographic composition would appear to be able to explain: across the nine standard regions composing England, depression incidence rates vary from 12 per 1000 (Eastern region) to 23.7 per 1000 (North West England). At MSOA level, standardized incidence ratios (new cases divided by expected new cases) vary from 0.13 to 3.97, with 5th and 95th percentiles of these ratios being 0.46 and 1.73, a nearly 4-fold contrast.

### 2.2. Potential Predictors for Regression Models

The choice of predictors for regression to explain such inequalities is based on evidence from other studies [3,6,7]. The first predictor is MSOA socio-economic status (SES). This index is based on UK Census 2021 indicators (see Appendix A). Area SES, and inverse status measures such as area deprivation, have been shown to influence levels of neighborhood mental ill-health [7].

Secondly, social fragmentation [35] is a measure of transience, household tenure insecurity, and a non-family household structure, which has been shown as a significant influence on neighbourhood rates of mental ill-health [7,35]. This index is also a composite based on Census data and has in some studies been taken as a proxy for neighborhood cohesion [36].

However, in the present study we develop a more direct index of neighborhood cohesion based on concepts underlying cohesion [37,38,39]. The methods used are set out in Section 2.3, and the derivation of this index precedes the regression analysis.

Many studies also show enhanced risk of mental ill health, including depression [40], in more urban areas, though measures of urbanicity vary. Here we adopt a measure based on concepts of urban form [41] (see Appendix A).

Crime levels are also associated with enhanced rates of mental ill-health [42], and crime may reinforce lower social cohesion. Violent crime and perceived lack of safety inhibit normal activities and may impact older people especially [9]. The study [9] mentions that for older people “characteristics of the environment [such as observed and perceived crime] assume greater salience” and criticizes previous studies of neighborhood effects, characterized by a “dominant trend of defining neighborhoods according to the socioeconomic status of its residents.”.

In the UK, ethnic mix is a potentially important influence on mental health outcomes. There is considerable evidence of higher psychosis among people of black and mixed ethnicity, but ethnic differentials in depression are less well researched. The study by Williams et al. [43] finds excess depression risk compared to white British groups, though the ethnicity effect is intertwined with that of socio-economic disadvantage and urbanicity.

### 2.3. Developing the Index of Neighborhood Social Cohesion: Cross-Scale Analysis

Our analysis includes developing a novel measure of small area (MSOA) neighborhood cohesion, of utility for public health needs mapping of social determinants. Spatial disaggregation is needed to estimate this cohesion index and involves three indicators, which are observed only at a higher spatial scale (296 local authorities). The indicators used are central criteria for cohesion [38], based on responses to questions in the Community Life Survey (CLS) of England [44], regarding belongingness, trust, and social interaction, specifically: how strongly do you feel you belong to your immediate neighborhood?; how often do you chat to your neighbors?; thinking about the people who live in this neighborhood, to what extent do you believe they can be trusted? We take as positive evidence of cohesion the responses: belonging ‘very strongly’ or ‘fairly strongly’; chatting more than once a month; and the answer that many of the people can be trusted.

The disaggregation estimation uses cross-scale methods [31,32], with the likelihood being for observed CLS data for England local authorities (e.g., on neighborhood belonginess), but based on a model predicting outcomes for MSOAs within local authorities. Bayesian methods are used via the BUGS program [45]; see Appendix B for a statistical specification. The neighborhood (MSOA) regression model includes neighborhood characteristics, based on evidence from the CLS regarding factors that especially impact on cohesion, and a neighborhood spatial random effect. The neighborhood cohesion index is then the score of the leading component from principal component analysis of the predicted proportions of MSOA populations with positive views of belongingness, of trust in neigbours, and of regular interaction with neighbours.

The characteristics used in the MSOA regression for the cross-scale models are area socio-economic status, proportions non-white, and urbanicity. For example, official commentary on CLS findings [46] reports that “adults living in rural areas were more likely to feel a sense of belonging to their neighborhood (69%) than adults living in urban areas (59%)”, while regarding interaction, “compared with the England average (69%), the proportion of adults who chatted to their neighbors at least once a month was higher among adults from the White British ethnic group (72%)”.

### 2.4. Initial Sifting of Predictors

We carry out initial regressions to sift out the most relevant predictors at MSOA level using a standard disease mapping approach within the R INLA program: spatially homogenous effects of area predictors, and a spatial (conditional autoregressive) random effect [11,12,24] to account for unmodelled spatial dependencies.

This approach is used because of collinearities between the predictors: between fragmentation and cohesion, and between cohesion and urbanicity. To anticipate later results, fragmentation and urbanicity are excluded from later analysis due to collinearities causing regression findings at odds with accumulated evidence.

### 2.5. Regression Strategy

With a reduced set of predictors, we consider four successive regression frameworks, the fit and implications of which we seek to assess. The first uses the standard disease mapping approach with a negative binomial likelihood in INLA, namely spatially homogenous predictor effects, no account of spatial spillover, and a spatial (conditional autoregressive) residual random effect.

The second introduces spatial lags for the environmental influences, crime and neighborhood cohesion, as additional predictors. We assume a spatial interaction matrix defined by adjacency, with $w_{ij}$ = 1 if MSOAs i and j are adjacent, and otherwise $w_{ij}$ = 0. Then the spatial lag calculation involves taking the average of the measure (say x) in neighborhoods adjacent to neighborhood i, namely $\sum_{j\neq i}w_{ij}x_{j}.$ This spatial lag calculation is provided by the R package spdep.

The third framework extends the second by taking spatially varying effects of the environmental variables (crime and cohesion), following the usual Bayesian spatially varying coefficient strategy [25]. This allows for possible non-stationary impacts of local environment variables, which may impact on their average effect.

The fourth is most general, allowing for region-specific heteroscedasticity both in spatially varying regression coefficients and in the spatial residual. The SVC strategy is applied to both the local and spillover environmental variables. We use the nine English standard regions to assess region-specific heteroscedasticity. This is implemented using the fbesag option within the R program INLA [26]; see Appendix C.

### 2.6. Spatial Regimes

Demonstrable non-stationarity from the regression stage may be summarized using the notion of spatial regimes. The R spatially constrained cluster analysis package ClustGeo, implements hierarchical clustering of area attributes, but also includes spatial constraints, so that spatial clustering in depression risk, neighborhood characteristics and predictor effects is allowed for. A mixing parameter is chosen to obtain both high intra-cluster homogeneity in neighborhood characteristics, but also high spatial proximity within clusters [47].

Spatial regimes offer a straightforward way of conveying to public health audiences differentiated patterns of disease incidence and associated causal influences. Thus the study by Myers et al. [48] reported (with regard to obesity in the US) that “associations between county-level adult obesity prevalence and county features differ between the regions of the US. […] Accordingly, this study holds implications for community-based obesity treatment and prevention efforts that apply a universal or one-size-fits-all approach to addressing the obesity epidemic”.

### 2.7. Measuring Goodness of Fit

We use three goodness of fit measures to compare these regression frameworks: the deviance information criterion (DIC) [49], the widely applicable information criterion (WAIC) [50], and the average absolute deviation (AAD) between actual and fitted incidence counts.

## 3. Results

### 3.1. Findings from Cross-Scale Modelling Regarding Neighborhood Cohesion

Table 1 shows the results of the three regressions which form part of the cross-scale modelling of neighborhood cohesion. It is evident from these regressions that less urban environments are significantly associated with a greater sense of neighborhood belongingness, with trusting neighbors, and with neighborhood social interaction. Higher neighborhood SES is also associated with higher levels of belongingness and trust. Higher proportions of non-white groups tend to reduce trust and neighborhood interactions.

The three MSOA indicators obtained from the cross-scale analysis are then combined into a single cohesion score via principal component analysis. The first component accounts for 87% of variation in the three separate indicators. This score is positive for areas with high cohesion.

The strong association of neighborhood cohesion with urban level apparent from Table 1 also shows if MSOAs are classified according to whether or not they are in the highest quintile for the resulting cohesion scores. Table 2 shows percentages of neighborhoods with high cohesion across the nine English region and according to a three-way urban settlement classification [51].

Across all regions, highest cohesion is most apparent in smaller rural settlements. By contrast, urban areas have low cohesion. Thus, only 3% of London neighborhoods exhibit high cohesion, and only 10% of all urban neighborhoods show high cohesion.

There is in fact a correlation of −0.87 between the urbanicity score and the cohesion score. Such collinearity is relevant to choice of predictors in subsequent regressions involving varying depression incidence as the outcome.

### 3.2. Multicollinearity

Initial regression of depression incidence following the standard disease mapping approach included the following neighborhood predictors: area SES; percent non-white; social fragmentation; urbanicity; social cohesion score; and crime level. This regression showed negative impacts of urbanicity and fragmentation, at odds with substantive evidence and reflecting collinearity: namely correlations of −0.87 between urbanicity and cohesion; of 0.61 between urbanicity and fragmentation; −0.54 between fragmentation and cohesion; and of 0.59 between crime and urbanicity. We therefore retain a subset of predictors with effects in this initial regression that are in line with substantive evidence, namely: area SES; percent non-white; cohesion and crime.

### 3.3. Neighborhood Variations in Depression Incidence: Regression Sequence

Table 3 shows the results of the regression sequence proposed in Section 2.5 with these four predictors—extending cohesion and crime effects to include spatial lags in models M2 to M4. The predictors are all on a [0, 1] scale, and the coefficients in Table 3 represent logged relative risks of depression incidence.

Table 3 shows the improved fit obtained under the second and subsequent models (denoted M2, M3 and M4), allowing for spillover and non-stationarity, as compared to the standard model (M1). The improved fit is especially notable for model M4 with region specific spatial precisions combined with varying spillover effects.

Regarding environmental impacts, comparing spillover and nonstationary models to the standard model, spillover and local effects of both socio-behavioral environments are shown to be cumulative. Both the local and spillover effects for cohesion in M2, M3 and M4 are negative, namely greater cohesion reduces depression incidence (a protective effect). Both the local and spillover effects of crime are positive, namely higher crime increases depression incidence. This cumulation of effects (local and spillover) enhances the overall role of these environments in explaining variations in depression incidence.

### 3.4. Comparing Relative Depression Risks

Accordingly, we consider implications for effects of cohesion and crime under different regression models. For an MSOA with high local crime, say 0.9 on the crime scale, and high spillover crime, also 0.9, the anticipated relative risk can be compared with an MSOA with low local crime, say 0.1, and low spillover crime, also 0.1. Other predictors are assumed to have value 0.5.

Model M2 adds spatial lag effects to the standard disease mapping approach. The spillover effect is most pronounced for crime, with coefficient of 0.306 and 95% interval (0.139, 0.473). This exceeds the local effect, which has a coefficient of 0.243. The major spillover effect of crime is maintained in model M3 and M4.

Considering the coefficients in M2, the relative depression risk for high crime areas is obtained as 1.24, compared to 0.80 in low crime areas, a relative risk ratio of 1.55. By contrast, under the standard model, M1, the contrast in these respective relative risks is attenuated: 1.10 vs. 0.88, or a ratio of 1.26.

Similarly, under M2, relative depression incidence for high cohesion areas is 0.849, as compared to relative incidence of 1.157 in low cohesion areas. So, the protective effect (reduced risk) can be expressed as a ratio of these two extremes, namely 0.73. In the standard model, this protective effect is closer to the default 1, namely 0.80.

### 3.5. Models with Non-Stationarity

It is notable that nonstationary models, both M3 and M4, show most evidence of non-stationarity for cohesion effects, as measured by the standard deviation of varying neighborhood coefficients around the average coefficient. The standard deviation in model M4 of varying cohesion coefficients is 0.215 (around the average coefficient of −0.219). Variation in the crime coefficients is much smaller, with a standard deviation of 0.016. In model M3 these two standard deviations are respectively 0.223 and 0.012.

**Figure 1:**
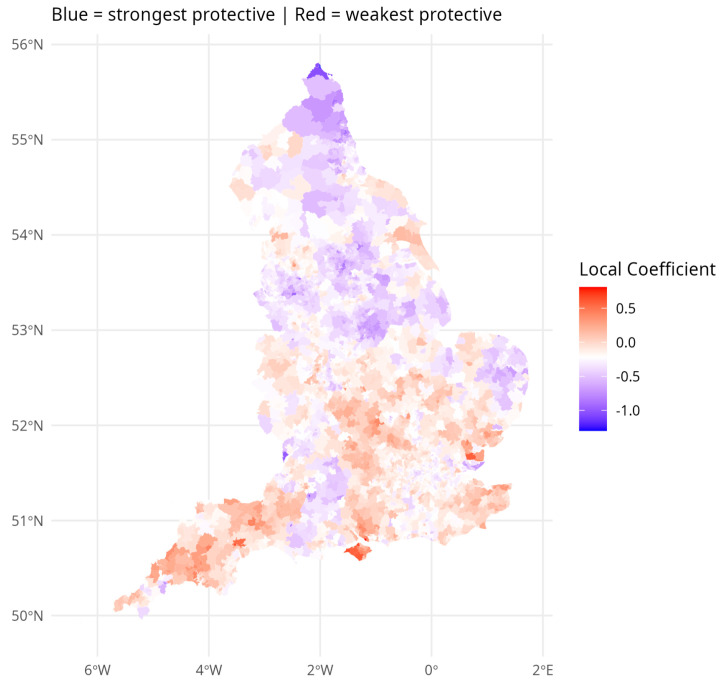
Varying Local Cohesion Regression Effect.

**Figure 2:**
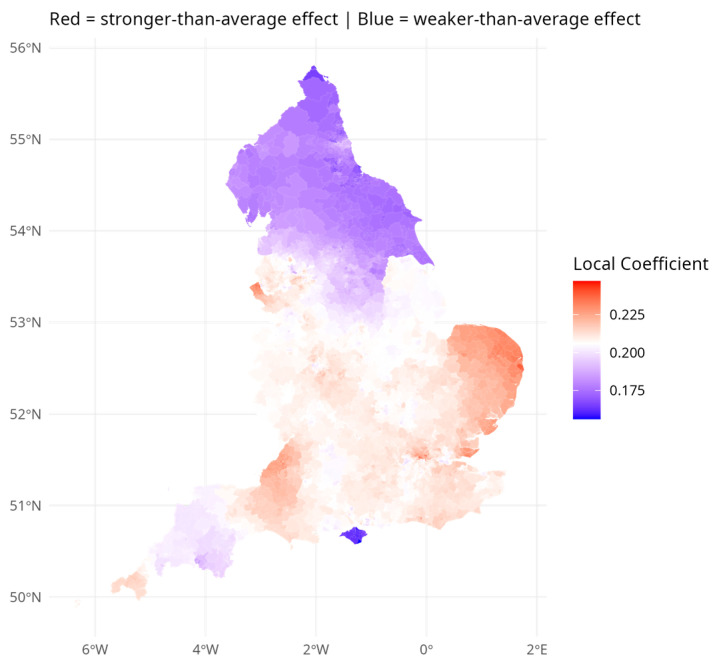
Varying Local Crime Regression Effect.

Figure 1 and Figure 2 show the varying coefficient estimates from model M4 for cohesion and crime respectively. The strongest—most negatively signed and hence most protective—effects of cohesion are in the northern half of England. By contrast, the strongest—most adverse—effects of crime on depression risk seem to be in the southern half of England. In the South East region outside London, mostly relatively prosperous and less urbanized, the average local cohesion coefficient is just −0.05, compared to the average −0.219. These patterns suggest that crime effects are stronger where cohesion effects are weaker—that cohesion moderates the impact of crime.

Figure 1 and Figure 2 demonstrate that environmental effects on depression are differentiated over broad regional settings. Table 4 quantifies these differences. Panel A in Table 4 reports fitted relative risks of depression incidence by region and settlement type, with England = 1.00 as the benchmark. Urban areas consistently show higher risk than rural areas within the same region, with the sharpest contrast in the North West (urban: 1.50 vs. smaller rural: 1.08). Panel B reports the average crime and cohesion effects within each region–settlement combination. The crime effect is relatively uniform across England (ranging from 0.17 to 0.22), whereas the cohesion effect varies markedly, from −0.41 in the North East to just −0.05 in the South East. In northern regions, cohesion is the dominant environmental influence on depression; in the South East, crime takes that role.

### 3.6. Implications for Spatial Regimes

These contrasting regional and urban-rural patterns recall the notion of spatial regimes discussed in Section 2.6. While Table 4 uses administrative regions, a spatial regime analysis groups neighborhoods by similarity in their characteristics and risk factor patterns. We apply ClustGeo with 12 clusters and mixing parameter 0.3, showing a moderate 13% loss in attribute homogeneity and a 15% loss in spatial homogeneity [49], p. 1815.

Table 5 presents three panels: Panel A shows cluster-level averages for neighborhood attributes and estimated effect sizes; Panel B shows how MSOAs are distributed across clusters by region; and Panel C shows the distribution by settlement type. Southern clusters (e.g., 4 and 12, concentrated in the South East and South West) have low urbanicity and show crime effects outweighing cohesion effects. Northern clusters (e.g., 6, 9, and 10) show the reverse: the protective cohesion effect outweighs the crime effect. Cluster 6 concentrated in the North East and Yorkshire/Humberside, exhibits the strongest cohesion effect (−0.44), nearly double the national average, and is predominantly urban, indicating that cohesion’s protective role in the North operates mainly in urban settings.

## 4. Discussion

There is extensive literature on the canonical disease mapping approach for disease counts, set out first by Besag et al. [24], and discussed in later reviews [11,12,52]. This specifies spatially constant effects of known neighborhood risk factors, no account for spillover in such risk factors, and with an omnibus spatially correlated residual to represent various unmodelled spatial dependencies.

The number of studies adapting this canonical approach to consider spatially varying regression coefficients or spatial volatility is, by contrast, relatively small, certainly much fewer than the number of frequentist studies applying GWR and linear model spatial volatility—for reviews of the latter see Comber et al. [53] and Otto et al. [29]. The lack of consideration of spatial heterogeneity in neighborhood disease studies is noted by Sridharan et al. [20] using a non-Bayesian form of spatial model. This neglect of possible non-stationarity may lead to a potentially oversimplified view of risk factor effects, and rules out the potential for identifying spatial regimes. This has parallel policy framing relevance: a one size fits all approach to priority setting is likely to similarly oversimplify and neglect spatially nuanced variations in environmental impacts, and by implication, spatially adaptive public health interventions.

Similarly, the great majority of disease mapping studies do not evaluate the extent of spillover in environmental risk factors, where environmental risks (crime, pollution, healthy food access, neighborhood social cohesion, etc.) are not defined by the arbitrary administrative boundaries most commonly used in disease mapping. However, acknowledging spillover is central to causal approaches to assessing neighborhood exposures. Spatial interactions are a form of network and relevant causal approaches acknowledging spillover (known as interference in the causal literature) are set out by Forastiere et al. [54]. From a substantive viewpoint, the notion of extended neighborhoods, suggested by Graif [17] in relation to spillover effects in neighborhood crime, is also relevant to environmental impacts on disease.

Without considering these two respects, non-stationarity and spillover, the canonical disease model can be considered as potentially simplistic, and may underestimate environmental impacts. This is certainly the case in the present study of neighborhood depression incidence, where we assess impacts of contextual neighborhood environments.

We have focused especially on environmental-contextual impacts on depression, similarly to the approach in [9], who criticize the over-emphasis on the deprivation-health link. To assist in pursuing this focus, we have developed a new measure of neighborhood cohesion, via spatial disaggregation. Our findings reinforce existing evidence [7,55,56] on impacts of cohesion and crime on depression, with the spatial regime findings showing the interplay of these influences: typically, one is weaker while the other is stronger.

In terms of modelling effectiveness, we find a considerably improved fit through acknowledging non-stationarity and spillover in environmental impacts. We have shown considerable variability in impacts of social environments, especially neighborhood cohesion. Spillover effects are also considerable, especially for neighborhood crime.

### Policy Relevance

Our study has therefore highlighted both the strength and spatial variability of crime and social cohesion effects. Spillover effects are also clearly relevant. Moreover, neighborhood cohesion measures have been problematic to measure directly, and this paper has shown their importance to mental health.

By considering commonly used approaches critically, our broader goal has been to establish that a more nuanced approach is relevant to gauging impacts of environmental determinants on mental health outcomes, so guiding public health intervention and needs assessment more precisely. As the study [48] mentions, a one-size-fits-all approach has limitations and (with regard to neighborhood obesity) that “continued research focusing on space and place in relation to obesity prevalence should further elaborate distinctive areas of the US in need of tailored interventions and public health policies and the unique factors linked to obesity across areas of the country”. A spatial regime analysis, as discussed above, translates and summarizes these varying influences in a way accessible to public health planners.

Designing place-based interventions to reduce crime and increase social cohesion should reflect subregional (e.g., urban–rural) differences in their impacts, and overspilling effects across boundaries. This implies gains from intersectoral collaboration (e.g., between public health, policing, urban planning, and community development) [57].

The contribution of the present paper is also to public health intelligence, a prerequisite to policy framing [58]. For example, our development of a neighborhood index of cohesion via spatial disaggregation has wider implications for improved neighborhood risk profiling. Thus, Rothenberg et al. [59], mention “advances in the ability to compare large areas, but with a concomitant deficiency in tools for public health workers to assess the status of local health and health disparities. Large area assessments are important, but the need for neighborhood action requires a greater focus on local information and analysis, emphasizing method over prespecified content”.

## 5. Conclusions

We have focused in this study on contextual effects in spatial epidemiology, namely the impacts of socio-behavioral environments on neighborhood mental health, and the implications for public health policy-framing. In particular, we have considered variations in neighborhood depression incidence according to levels of cohesion and crime, and aimed to provide a nuanced assessment of how these impacts operate spatially, moving beyond the simplifying assumptions of the standard Bayesian disease mapping approach. We have shown much improved fit through acknowledging non-stationarity and spillover, apparent especially in variable impacts of neighborhood cohesion, and spillover effects for neighborhood crime.

## Figures and Tables

**Table 1:** Cross-Scale Neighborhood Regression Results.

Log odds ratio coefficients (posterior means with 95% credible intervals) from cross-scale models predicting MSOA-level neighbourhood cohesion indicators. Negative values for urbanicity indicate that less urban areas show higher cohesion.			
Sense of Belonging			
	Mean	2.5%	97.5%
Area SES	0.49	0.21	0.73
Proportion Non-white	−0.22	−0.40	0.02
Urbanicity	−1.35	−1.73	−0.99
Many Neighbors can be Trusted			
	Mean	2.5%	97.5%
Area SES	2.78	2.56	3.07
Proportion Non-white	−0.85	−1.14	−0.63
Urbanicity	−1.90	−2.29	−1.40
Chat with Neighbors More than Once a Month			
	Mean	2.5%	97.5%
Area SES	−0.12	−0.38	−0.12
Proportion Non-white	−0.34	−0.51	−0.34
Urbanicity	−2.74	−3.05	−2.75

**Table 2:** Percentages of Neighborhoods (MSOAs) with High Cohesion by Region and Urban Level.

High cohesion is defined as falling in the top 20% of MSOAs nationally on the composite cohesion score derived from cross-scale modelling (Section 2.3). A dash (-) indicates the settlement type does not apply. Settlement classification follows the ONS 2021 Rural–Urban Classification.				
	Settlement Category			
Region	Larger rural	Smaller rural	Urban	All Categories
East Midlands	47	76	9	22
Eastern England	34	66	11	22
London	-	-	3	3
North East	24	100	11	15
North West	66	95	15	21
South East	55	91	15	25
South West	54	99	12	33
West Midlands	63	98	9	19
Yorkshire-Humber	64	100	11	21
All of England	50	88	10	20

**Table 3:** Predicting Neighbourhood Depression Incidence.

Coefficients Represent Logged Relative Risks with Predictors on [0, 1] scale					
Coefficients are posterior means of logged relative risks (with standard deviations and 95% credible intervals). Models M1–M4 are cumulative extensions: M1 is the baseline (stationary effects, no spillover); M2 adds spatial lags for cohesion and crime; M3 adds spatially varying coefficients for environmental predictors; M4 further allows region-specific spatial precisions (flexible Besag). Fit Measures: DIC = deviance information criterion; WAIC = widely applicable information criterion; MAD = mean absolute deviation between observed and fitted incidence counts.					
Predictor	Statistic	M1	M2	M3	M4
Intercept	Mean	0.039	0.012	0.028	0.034
	St devn	0.045	0.095	0.094	0.095
	(2.5%, 97.5%)	(−0.050, 0.127)	(−0.174, 0.198)	(−0.156, 0.211)	(−0.152, 0.220)
Area SES	Mean	−0.149	−0.192	−0.150	−0.249
	St devn	0.042	0.043	0.042	0.041
	(2.5%, 97.5%)	(−0.231, −0.066)	(−0.276, −0.108)	(−0.231, −0.068)	(−0.329, −0.169)
Proportion Non-white	Mean	0.031	−0.013	−0.044	−0.089
	St devn	0.038	0.039	0.038	0.038
	(2.5%, 97.5%)	(−0.043, 0.106)	(−0.089, 0.064)	(−0.118, 0.031)	(−0.162, −0.015)
Crime Index	Mean	0.285	0.243	0.242	0.206
	St devn	0.040	0.040	0.040	0.040
	(2.5%, 97.5%)	(0.207, 0.363)	(0.165, 0.322)	(0.163, 0.321)	(0.128, 0.284)
Neighbourhood Cohesion	Mean	−0.276	−0.191	−0.274	−0.219
	St devn	0.053	0.054	0.053	0.052
	(2.5%, 97.5%)	(−0.379, −0.173)	(−0.297, −0.086)	(−0.378, −0.169)	(−0.321, −0.118)
Crime Spillover	Mean	—	0.306	0.300	0.309
	St devn	—	0.085	0.084	0.085
	(2.5%, 97.5%)	—	(0.139, 0.473)	(0.135, 0.465)	(0.143, 0.475)
Cohesion Spillover	Mean	—	−0.195	−0.178	−0.104
	St devn	—	0.082	0.079	0.079
	(2.5%, 97.5%)	—	(−0.356, −0.035)	(−0.334, −0.022)	(−0.259, 0.051)
Model Fit					
DIC		58,647	58,519	58,010	56,307
WAIC		58,173	58,098	57,431	55,359
Mean Absolute Deviation		4.916	4.882	4.415	3.334

**Table 4:** Regional Patterns of Risk and Risk Factor Impacts. (Standard Regions and Settlement Types across England). Panel A: Fitted relative risks of depression incidence by region and settlement type, benchmarked to the England average (1.00). Values above 1.0 indicate elevated risk. Panel B: Estimated local effect sizes for crime (positive = higher crime increases depression) and cohesion (negative = greater cohesion reduces depression) from model M4 spatially varying coefficients. A dash (—) indicates the settlement type does not apply to the region.

(A)								
Region	Larger Rural		Smaller Rural		Urban		All Settlement Types	
East Midlands	0.79		0.77		0.84		0.82	
East of England	0.75		0.64		0.76		0.74	
London	—		—		0.93		0.93	
North East	0.95		0.72		1.10		1.07	
North West	1.12		1.08		1.50		1.46	
South East	0.99		1.00		1.06		1.05	
South West	0.76		0.76		0.87		0.84	
West Midlands	1.04		0.93		1.17		1.15	
Yorks/Humberside	0.74		0.66		0.90		0.87	
England	0.86		0.82		1.03		1.00	
(B)								
Region	Larger Rural		Smaller Rural		Urban		All Settlement Types	
	Crime	Cohesion	Crime	Cohesion	Crime	Cohesion	Crime	Cohesion
East Midlands	0.20	−0.26	0.20	−0.26	0.20	−0.30	0.20	−0.29
East of England	0.22	−0.12	0.22	−0.20	0.21	−0.16	0.22	−0.16
London	—	—	—	—	0.21	−0.23	0.21	−0.23
North East	0.17	−0.40	0.17	−0.48	0.18	−0.41	0.18	−0.41
North West	0.19	−0.26	0.19	−0.24	0.21	−0.32	0.20	−0.31
South East	0.21	−0.04	0.21	−0.04	0.21	−0.06	0.21	−0.05
South West	0.21	−0.10	0.21	−0.10	0.21	−0.17	0.21	−0.15
West Midlands	0.21	−0.15	0.21	−0.16	0.21	−0.17	0.21	−0.17
Yorks/Humberside	0.19	−0.35	0.18	−0.31	0.19	−0.39	0.19	−0.38
England	0.20	−0.19	0.21	−0.16	0.21	−0.23	0.21	−0.22

**Table 5:** Cluster Characteristics and Regional Distribution. Twelve clusters are obtained from spatially constrained hierarchical clustering (ClustGeo, mixing parameter = 0.3). Panel A: Mean values of neighbourhood attributes and estimated effect sizes from model M4 for each cluster; the final column (All) shows the national average. Panel B: Number of MSOAs assigned to each cluster within each English region. Panel C: Number of MSOAs by settlement type. Clusters are numbered 1–12 (C1–C12) in the order produced by the algorithm.

(A)													
Attribute	C1	C2	C3	C4	C5	C6	C7	C8	C9	C10	C11	C12	All
Depression Incidence Ratio	0.91	0.76	1.09	0.99	1.01	0.95	1.61	1.04	0.74	1.32	0.56	0.98	1.00
Neighbourhood Cohesion	0.38	0.65	0.55	0.73	0.70	0.56	0.52	0.34	0.73	0.33	0.66	0.66	0.58
Neighbourhood Crime	0.59	0.41	0.52	0.37	0.39	0.60	0.61	0.61	0.40	0.61	0.40	0.43	0.49
Fragmentation Index	0.32	0.15	0.21	0.12	0.14	0.22	0.23	0.21	0.16	0.64	0.18	0.21	0.21
Area SES	0.55	0.69	0.64	0.78	0.71	0.50	0.48	0.34	0.71	0.60	0.67	0.65	0.62
Non-white Proportion	0.46	0.10	0.22	0.09	0.07	0.11	0.15	0.65	0.04	0.30	0.06	0.05	0.18
Urbanicity Index	0.54	0.34	0.41	0.30	0.33	0.40	0.42	0.49	0.34	0.53	0.35	0.35	0.39
Local Crime Effect	0.21	0.21	0.21	0.21	0.21	0.19	0.21	0.20	0.18	0.20	0.20	0.21	0.21
Local Cohesion Effect	−0.23	−0.14	−0.09	−0.05	−0.30	−0.44	−0.21	−0.32	−0.35	−0.37	−0.28	−0.03	−0.22
Spillover Crime Effect	0.31	0.30	0.31	0.31	0.31	0.30	0.31	0.31	0.30	0.31	0.31	0.32	0.31
Cohesion Spillover Effect	−0.10	−0.10	−0.10	−0.10	−0.10	−0.12	−0.10	−0.11	−0.12	−0.11	−0.11	−0.11	−0.10
(B)													
Region	C1	C2	C3	C4	C5	C6	C7	C8	C9	C10	C11	C12	All
E Midl	30	131	13	6	199	105	31	30	14	16	0	0	575
East of England	132	544	53	9	7	0	0	0	0	1	0	0	746
London	683	25	250	44	0	0	0	0	0	0	0	0	1002
North East	0	0	0	0	2	286	0	6	38	10	0	0	342
North West	0	0	0	0	348	39	424	38	55	28	0	0	932
South East	65	77	447	461	5	0	3	0	0	1	60	0	1119
South West	22	0	3	0	13	0	0	2	0	0	242	428	710
West Midlands	8	29	6	3	286	3	244	130	0	12	15	0	736
Yorks/Humberside	0	2	0	0	17	231	69	33	318	24	0	0	694
(C)													
Settlement Type	C1	C2	C3	C4	C5	C6	C7	C8	C9	C10	C11	C12	All
Larger Rural, Further	0	36	2	8	26	9	2	0	16	0	23	34	156
Larger Rural, Closer	1	104	12	53	98	39	8	0	61	0	13	22	411
Smaller Rural, Further	0	46	0	18	45	5	0	0	27	0	25	68	234
Smaller Rural, Closer	0	73	4	83	92	4	0	0	28	0	17	24	325
Urban, Further	18	88	42	20	20	10	24	0	38	2	52	87	401
Urban, Closer	921	461	712	341	596	597	737	239	255	90	187	193	5329

## Data Availability

Data and code are provided at Harvard Dataverse.

## Appendix A. Data Sources for Predictor Variables

Data for predictors are drawn primarily from the 2021 UK Census but with some non-Census data. The index of urbanicity is based on a principal component analysis of five indicators: population density (in relation to total MSOA land area); population residential density (in relation to MOSA residential land area); distance to a primary care practice; proportions of open land; and greenspace access, specifically the proportion of greenspace within a 900 metre buffer from people’s homes. These indicators are based on the 2021 Census; on the Access to Healthy Assets and Hazards dataset (https://www.cdrc.ac.uk/); and England Land Use 2022 statistics (https://www.gov.uk/government/statistics/land-use-in-england-2022/land-use-statistics-england-2022; accessed on 1 June 2025).

Neighborhood socio-economic status (SES) is obtained from principal component analysis of three 2021 Census indicators: proportions of economically active professional and managerial; proportions of households not deprived on any dimension (Census Table TS011, Households by Deprivation Dimensions); and the unemployment rate.

Social fragmentation is obtained from principal component score of four 2021 Census indicators: residential turnover in the year 2020–2021; one person households; renting from private sector landlords (“private renting”); and non-partnered adults (not married or in civil partnership).

The crime index is from the 2019 Index of Multiple Deprivation (IMD) (https://opendatacommunities.org/data/societal-wellbeing/imd2019/indices; accessed on 1 May 2025) and measures the risk of personal and material victimization based on indicators recorded violent crimes, burglaries, thefts, and criminal damage.

## Appendix B. Cross-Scale Modelling Specification

The observed data for cross-scale modelling consist of total positive responses and total survey respondents {$Y_{j}$,$P_{j}\}\;$ for higher level spatial units, which are M = 296 English local authorities. The cross-scale model framework involves regression to predict positive response rates for N = 6856 lower level spatial units, followed by cumulation over these units to predict local authority rates. As mentioned in the text, the lower level units are Middle Level Super Output Areas (MSOAs). Let $J_{i}=j$ denote the local authority to which MSOA *i* = 1, …, N belongs, and let $w_{i}$ denote the share of MSOA *i* in the population of local authority *j* (with these shares summing to 1 within each local authority).

Then assume a binomial likelihood at region level, $Y_{j}$~Bin($P_{j},R_{j}$), where $R_{j}$ is the local authority probability of a positive outcome (belonging, trusting, chatting). The lower scale regression model for MSOAs involves predictors $X_{i}$, a spatial random effect $s_{i}$ at MSOA level, here a conditional autoregressive model [27], and a local authority level iid random error *u*$\left(J_{i}\right)$. This model predicts MSOA level event rates $r_{i}$, with the region random effects ensuring that local authority event totals $Y_{j}$ are closely reproduced.

The MSOA model, with a logit link, is then

logit(*r_i_*) = α + *X_i_*β + *s_i_* + *uJ_i_*,

and predicted region level probabilities $R_{j}$ are obtained by summing over the $r_{i}$ within the relevant local authority, namely with $J_{i}=j$, weighted according to their population shares $w_{i}$ within that local authority.

## Appendix C. Non-Stationary Variance Specification: The Flexible Besag

The study [26] adapts the model of Besag et al. [27] to provide the flexible Besag (fbesag) approach. The study [26] assumes subregion-specific smoothness for a single latent effect, namely the spatially varying intercept. Here we extend that framework to spatially varying regression coefficients.

The stationary model of [27] assumes a single precision π. The flexible Besag instead assumes each subregion *k* has its own smoothness: larger $\pi_{k}$ produce stronger smoothing; smaller $\pi_{k}$ represent more local variability. We parameterize $\pi_{k}$ = πexp($\gamma_{k}$), and place fbesag priors on deviations of spatially varying coefficients around the overall fixed effect, producing nonstationary SVC surfaces. When all $\pi_{k}$ are equal, the model reduces to the stationary model.

Consider for simplicity, the case of a single spatially varying coefficient for predictor $x_{i}$, without a spatially varying intercept. Then consider a negative binomial outcome $y_{i}$ ∼NB($\mu_{i},\phi )$, with ϕ a dispersion parameter, and $\mu_{i}=E_{i}\rho_{i}$ with $E_{i}$ denoting expected cases, and $\rho_{i}$ denoting relative risks in neighborhood i. Further let $\tau_{i}$ denote neighborhood spatial precisions, $R_{i}$ denote the regions to which neighborhoods *i* belong to, and in the equations below let $l=R_{i}$, and m = $R_{j}$ for neighborhoods j adjacent to neighborhood i. Also let $n_{im}$ be the number of neighborhoods adjacent to neighborhood i in region m, and $n_{i}=\sum_{m}n_{im}$.

Then the relative risk model under the fbesag prior is

$$log(\rho_{i})=\beta_{0}+\left(\beta_{1}+u_{i}\right)x_{i},$$

where $\beta_{1}$ is a fixed effect coefficient, the $u_{i}$ are spatially correlated random effects constrained to sum to zero, and the conditional prior for the $u_{i}$ is

$$u_{i}\;∼\;N(0.5\sum_{j\neq i}(\pi_{l}+\pi_{m})u_{j}/\tau_{i},1/\tau_{i}),$$

where neighborhood precisions are defined as

$$\tau_{i}\;=\;{0.5(n}_{i}\pi_{l}+\sum_{m}n_{im}\pi_{m}).$$

This specification reduces to the Besag et al. model [27] when all sub-region precisions are equal.

In model M4 of Table 2, we in fact assume four spatially varying coefficients to which the fbesag prior is applied. We also keep an fbesag prior for a spatially varying intercept. We partition the MSOAs into K groups, namely the K = 9 England standard regions, but other partitions are possible.

To discourage overfitting and ensure contraction to the stationary Besag [27] as a base model, we follow the penalizing complexity (PC) prior framework of Simpson et al. [28]. For a Gaussian latent component with standard deviation σ, the PC prior places an exponential penalty on σ,

f(σ) = λexp⁡(−λσ), σ > 0

which induces the precision-scale form

$$f\left(\pi \right)=(\lambda /2)\pi^{-3/2}\;exp{(-\lambda \pi }^{-0.5}),\;\pi >0$$

with calibration Pr(σ > u) = α, giving

λ = −log(α)/u, u > 0.

Let θ = (log $\pi_{1}$, …, log $\pi_{K}$), with mean $\underset{-}{\theta }$ = (1/*K*)$\sum_{k}\theta_{k}$ and $\hat{\Sigma }$ = $s^{2}$(I − (1/*K*)$11^{T}$), where s is the flexibility parameter of γ’s, I is an identity matrix of dimension *K*, and 1 is a vector of ones. Then, the joint PC prior of θ may be stated as

$$f(\theta )\propto \exp{\left(-0.5(\theta -\underset{-}{\theta }1)\right)}^{T}\;\;{\hat{\Sigma }}^{-1}(\theta -\underset{-}{\theta }1)-0.5\underset{-}{\theta }-\lambda \exp(-0.5\underset{-}{\theta }).$$

The λ and s can be selected by the user based on some prior knowledge. The small s enforces near-stationarity (γ_*k*_ ≈ 0), while larger values allow substantively meaningful regional heterogeneity; see [26] for a detailed discussion of suitable values.
